# The role of strigolactone in alleviating salinity stress in chili pepper

**DOI:** 10.1186/s12870-024-04900-4

**Published:** 2024-03-23

**Authors:** Subhan Danish, Misbah Hareem, Khadim Dawar, Tayyaba Naz, Muhammad Mazhar Iqbal, Mohammad Javed Ansari, Saleh H. Salmen, Rahul Datta

**Affiliations:** 1 Department of Soil Science, Faculty of Agricultural Sciences and Technology, Bahauddin Zakariya University, Multan, Punjab Pakistan; 2 Department of Environmental Sciences, Woman University Multan, Multan, Punjab Pakistan; 3 Department of Soil and Environmental Science, the University of Agriculture Peshawar, Peshawar, Pakistan; 4 Saline Agriculture Research Centre, Institute of Soil and Environmental Sciences, University of Agriculture Faisalabad, Faisalabad, 38400 Pakistan; 5 Department of Soil and Environmental Sciences, College of Agriculture, University of Sargodha, Sargodha, 40100 Pakistan; 6 Department of Botany, Hindu College Moradabad (MJP Rohilkhand University Bareilly), Moradabad, 244001 India; 7 Department of Botany and Microbiology, College of Science, King Saud University, PO Box -2455, Riyadh, 11451 Saudi Arabia; 8 Department of Geology and Pedology, Faculty of Forestry and Wood Technology, Mendel University in Brno, Zemedelska 1, Brno, 61300 Czech Republic

**Keywords:** Antioxidant, Chili chlorophyll content, Salinity stress, Strigolactone

## Abstract

Salinity stress can significantly delay plant growth. It can disrupt water and nutrient uptake, reducing crop yields and poor plant health. The use of strigolactone can be an effective technique to overcome this issue. Strigolactone enhances plant growth by promoting root development and improvement in physiological attributes. The current pot study used strigolactone to amend chili under no salinity and salinity stress environments. There were four treatments, i.e., 0, 10µM strigolactone, 20µM strigolactone and 30µM strigolactone. All treatments were applied in four replications following a completely randomized design (CRD). Results showed that 20µM strigolactone caused a significant increase in chili plant height (21.07%), dry weight (33.60%), fruit length (19.24%), fruit girth (35.37%), and fruit yield (60.74%) compared to control under salinity stress. Significant enhancement in chili chlorophyll a (18.65%), chlorophyll b (43.52%), and total chlorophyll (25.09%) under salinity stress validated the effectiveness of 20µM strigolactone application as treatment over control. Furthermore, improvement in nitrogen, phosphorus, and potassium concentration in leaves confirmed the efficient functioning of 20µM strigolactone compared to other concentrations under salinity stress. The study concluded that 20µM strigolactone is recommended for mitigating salinity stress in chili plants. Growers are advised to apply 20µM strigolactone to enhance their chili production under salinity stress.

## Introduction

Salinity is a prominent abiotic stress factor with far-reaching implications for global crop productivity [1–6]. It adversely affects germination and overall plant vigor, resulting in substantial crop losses worldwide. Around 20–33% of the world’s total land area, including irrigated lands, face the impact of salinity issues. Projections suggest that this percentage could increase to 50% by 2050 [1, 7]. This is particularly concerning as many economically valuable crops as possible, notably horticultural ones, display sensitivity to salinity.

Salinity stress also leads to the accumulation of reactive oxygen species (ROS) in plant cells, causing potassium (K^+^) efflux from the cells. Elevated ROS levels weaken the plant’s defense mechanisms, leading to oxidative stress. ROS, when present in high concentrations, can cause substantial harm to plants. Under salinity stress, ROS levels increase and threaten plant cells by initiating processes like lipid peroxidation, protein oxidation, damage to nucleic acids, inhibition of enzyme activation, and cell death [8].

Strigolactone serves as a critical factor for improving crop resilience during stress [9, 10]. It can achieve this by fostering essential root development empowering plants to delve deeper into the soil layers in search of water and nutrients [11]. Strigolactone can also regulate stomatal closure, a crucial mechanism that minimizes water loss through transpiration while maintaining the necessary gas exchange for photosynthesis [12]. Additionally, strigolactone enhances overall stress tolerance by producing stress-related proteins and compounds. This activation significantly enhances plant resilience, enabling them to endure and thrive in challenging environmental conditions [10].

Chili (*Capsicum annuum* L.) is a cash crop which is widely cultivated due to its nutritional value [13]. It holds a significant position among vegetable crops and is an essential spice plant within the Solanaceae family. This crop is primarily cultivated for its spicy fruits, which are used in their ripe and green forms to enhance the flavor of various dishes. Green chilies are valued for their richness of vitamins A, C, minerals, and protein. In contrast, dried chili is known for its high vitamin A and D content, contributing to its characteristic hotness and spiciness in culinary applications [14, 15]. However, salinity stress significantly reduces chili production by triggering alterations in various physiological processes that delay plant growth and overall productivity. Salinity stress creates conditions of increased osmotic potential, resulting in a water deficit in the soil [16]. Additionally, it induces ionic toxicity by elevating the concentrations of harmful ions such as sodium (Na^+^) and chloride (Cl^-^) within the plant, causing an imbalance of essential nutrients within plant cells [16].

That’s why the current study aims to explore the potential of strigolactone to mitigate the effects of salinity stress on chili plants. Strigolactone has shown potential in maintaining ion homeostasis in plant roots. It can also improve water and nutrient acquisition in different crops, making them a valuable resource in salinity stress [17, 18]. The current study covers the knowledge gap regarding selecting the best application rate of strigolactone for chilies to mitigate salinity stress. We hypothesized that applying strigolactone can help alleviate the adverse effects of salinity stress on chilies and enhance their growth and productivity. The study’s core aim was to select the best strigolactone application rate for achieving better chilies growth when cultivated in salt-affected soils.

## Materials and methods

### Experimental site

In the year 2021, a pot experiment was carried out in the research site located at the Faculty of Agricultural Sciences and Technology, Bahauddin Zakariya University, situated in Multan, Punjab, Pakistan, with geographical coordinates of 30°15′49″N and 71°30′35″E. The study followed a completely randomized design (CRD) with four replicates. The physiochemical characteristics of soil and irrigation water are provided in Table 1.

**Table 1:** Pre-experimental soil and irrigation characteristics

Soil	Values	References	Irrigation	Values	References
pH	819	[19]	pH	7.26	[20]
EC*e* (dS/m)	2.42	[21]	EC (µS/cm)	387	
SOM (%)	0.60	[22]	Carbonates (meq./L)	0.00	
TN (%)	0.03	[23]	Bicarbonates (meq./L)	4.69	
AP (µg/g)	6.27	[24]	Chloride (meq./L)	0.05	
EK (µg/g)	127	[25]	Ca + Mg (meq./L)	2.69	
ENa (µg/g)	116	[26]	Sodium (mg/L)	106	
Texture	Clay Loam	[27]			

TN = Total Nitrogen; AP = Available Phosphorus; EK = Extractable Potassium; ENa = Extractable Sodium

### Pot preparation and sowing

A plastic pot (width = 15 inches and depth = 45 inches) was filled with 10 kg of soil. In each container, 20 seeds were sown, and, following a period of 21 days from germination, six healthy seedlings were retained after thinning.

### Fertilizer

To address the chili nutritional requirements, 25 kg per acre (0.31 g/10 kg soil) of nitrogen (N) and 12 kg per acre (0.15 g/10 kg soil) of phosphorus (P) were applied, utilizing urea as the nitrogen source and single superphosphate for phosphorus, at the recommended rates. Additional potassium (K) supplementation was added at the rate of 12 kg per acre (0.15 g/10 kg soil) using potassium sulfate.

### Irrigation

Irrigation for each pot was consistently monitored and adjusted using a moisture gauge (ADVANCED™; 4 in 1 Soil Meter; China). Daily observations were made to ensure that the moisture level was maintained at the designated threshold, with wet corresponding to 70% of the soil’s field capacity, as indicated by the instrument’s scale.

### Collecting, sterilization and sowing of seeds

The chili seeds used in this study were of Ghotki variety and were procured from a licensed seed merchant authorized by the Government of Punjab, Pakistan. Before sowing, a rigorous surface sterilization protocol was conducted on the selected seeds. This included rinsing the seeds three times with 95% ethanol after they had been exposed to 5% sodium hypochlorite solution. Subsequently, the seeds underwent three additional rinses using sterilized deionized water to eliminate any traces of the sterilizing agents. Each pot, filled with 5 kg of soil, initially received 20 seeds for sowing. After germination, a careful thinning process was performed, resulting in the retention of 10 seedlings in each pot (Ahmad et al., 2014).

### Strigolactone

Strigolactone GR24 was purchased from a certified SIGMA dealer in Multan. The details of the product include strigolactone GR24; CAS Number: 76974-79-3; MDL number: MFCD12405021.

### Salinity stress

A 1:1:1 mixture of NaCl, MgSO_4_, and CaCl_2_ was added to the soil with EC 2.34 dS/m (no salinity stress) to examine the effect of salinity stress. The final EC was maintained at 6.13 dS/m (salinity stress) by incubating the soil for 21 days before the start of the experiment. During the soil incubation, mixing was done regularly using a spatula so the salts may get homogenized in the soil.

#### Treatments

The chili seedlings were subjected to different strigolactone concentrations as a foliar spray when seedlings were 21 days old. The levels of strigolactone include 0 µM, 10µM, 20µM, and 30µM strigolactone. All the levels were applied under no salinity and salinity stress conditions.

### Harvesting and data collection

A total of 2 harvestings were performed. The first harvesting includes the collection of fresh leaves after 50 days of transplantation (at vegetative stage) for the analysis of chlorophyll contents, proline, antioxidants and other biochemical analysis [28]. However, after 85 days of transplantation chilies picking started. For assessment of yield total 8 picks were taken [28]. For the assessment of yield data, a total of 8 picks were taken. The study encompassed the examination of various parameters, including the total plant dry weight (g/plant), plant height (cm), the number of primary branches per plant, fruit length (cm), fruit girth (cm), fruit yield (kg/plant), and the assessment of chlorophyll and proline content.

### Estimation of Chlorophyll

In the research study, 0.5 mg of freshly harvested leaf samples were ground in a mortar and pestle with 20 ml of 80% acetone. Subsequently, mixture was subjected to centrifugation at 3000 rpm for 15 min, yielding a supernatant that was collected. The pellet was successfully treated with 5 ml of 80% acetone until it lost color. All the collected supernatants were combined for the determination of chlorophyll content. The measurement of absorbance at 645 and 663 nm was carried out using a spectrophotometer [29], and the chlorophyll content was quantified employing the following formulae.

$${\text{Chlorophyll}}\,{\text{a}}\left( {\frac{{{\text{mg}}}}{{\text{g}}}} \right) = \frac{{\left( {12.7 \times {\text{A}}663} \right) - \left( {2.69 \times {\text{A}}645} \right) \times {\text{V}}}}{{1000 \times {\text{W}}}}$$

$${\text{Chlorophyll}}\,{\text{b}}\left( {\frac{{{\text{mg}}}}{{\text{g}}}} \right) = \frac{{\left( {22.9 \times {\text{A}}645} \right) - \left( {4.68 \times {\text{A}}663} \right) \times {\text{V}}}}{{1000 \times {\text{W}}}}$$

$${\text{Total}}\,{\text{Chlorophyll}}\left( {\frac{{{\text{mg}}}}{{\text{g}}}} \right) = \frac{{20.2\left( {{\text{A}}645} \right) + 8.02\left( {{\text{A}}663} \right) \times {\text{V}}}}{{1000 \times {\text{W}}}}$$

### Antioxidants

For the analysis of antioxidants, 8th to 12th leaves above the ground were collected after 60 days of germination. We assessed superoxide dismutase (SOD) activity by measuring the inhibition of nitro blue tetrazolium (NBT) reduction in the presence of riboflavin [30]. The reaction mixture consisted of enzyme extract, NBT, riboflavin, and phosphate buffer, which was exposed to light, and changes in absorbance at 560 nm were monitored. Additionally, we determined the decrease in absorbance at 240 nm resulting from hydrogen peroxide (H_2_O_2_) decomposition, as per the method outlined by Aebi [31]. For ascorbate peroxidase (APX) activity, we tracked ascorbate oxidation in the presence of H_2_O_2_, following the procedure by Nakano and Asada [32], measuring the absorbance change at a specific wavelength over time. To assess lipid peroxidation, we quantified the malondialdehyde (MDA, an indicator of lipid peroxidation) by reacting the sample extract with thiobarbituric acid (TBA) to generate a colored complex. Then, we measured the absorbance of the complex to calculate the MDA content.

### Electrolyte leakage and proline

Initially 1 cm diameter leaves discs (1 g) were taken in 10 ml deionized water containing test tubes. After that test tubes were incubated at 25 °C for 24 h and electrical conductivity (EC1) was taken using EC meter. The test tubes were then subjected to a 20-minute heating (at 120 °C) in a water bath, and then second electrical conductivity measurement (EC2) was recorded [33].

$${\text{Electrolyte}}\,{\text{Leakage}}\left( \% \right) = \left( {\frac{{{\text{EC}}1}}{{{\text{EC}}2}}} \right) \times 100$$

### Proline determination

The quantification of proline in leaf samples was analyzed following the procedure outlined by Bates et al. [34]. The absorbance was measured at 520 nm using a Shimadzu UV 1601 spectrophotometer.

### Fruit harvest, dry weight, and nutrient analysis

For morphological data collection, three samples were randomly chosen from each treatment and subsequently separated into leaves, stems, and roots. These plant parts were then dried in an oven at 70 ± 8 °C for two days to establish their dry weights and elemental concentrations. All nutrient analyses were conducted on a dry-weight basis. The Kjeldahl method determined Total nitrogen content in 0.1 g samples of dry weight. Subsequently, ground samples were subjected to dry ashing at 500 ± 8 °C for 26 h, followed by mixing with 2 M hot HCl, filtration, and dilution to a final volume of 50 ml with distilled water.

### N, P, and K leaves

In this research study, nitrogen content was determined using a modified micro-Kjeldahl method as outlined by [35]. Potassium content analysis was performed through a flame photometer (Jenway PFP 7, Essex, England). Additionally, phosphorus content was quantified at 420 nm utilizing a spectrophotometer based on the vanadate molybdate method, following the procedure described by [35]. Sodium and potassium concentrations were assessed in these sample solutions. Phosphorus levels were analyzed using the vanadate molybdate method with a UV/Visible spectrophotometer (Shimadzu UV, 1601) [35].

### Statistical analysis

Conventional statistical procedures were employed to analyze the data, including the utilization of a two-way ANOVA to evaluate the significance of treatments. Paired comparisons were conducted using the Tukey test with a significant level set at *p* ≤ 0.05. Pearson correlations were performed by the OriginPro software [36].

## Results

### Plant height, plant dry weight and number of primary branches

**Fig. 1:**
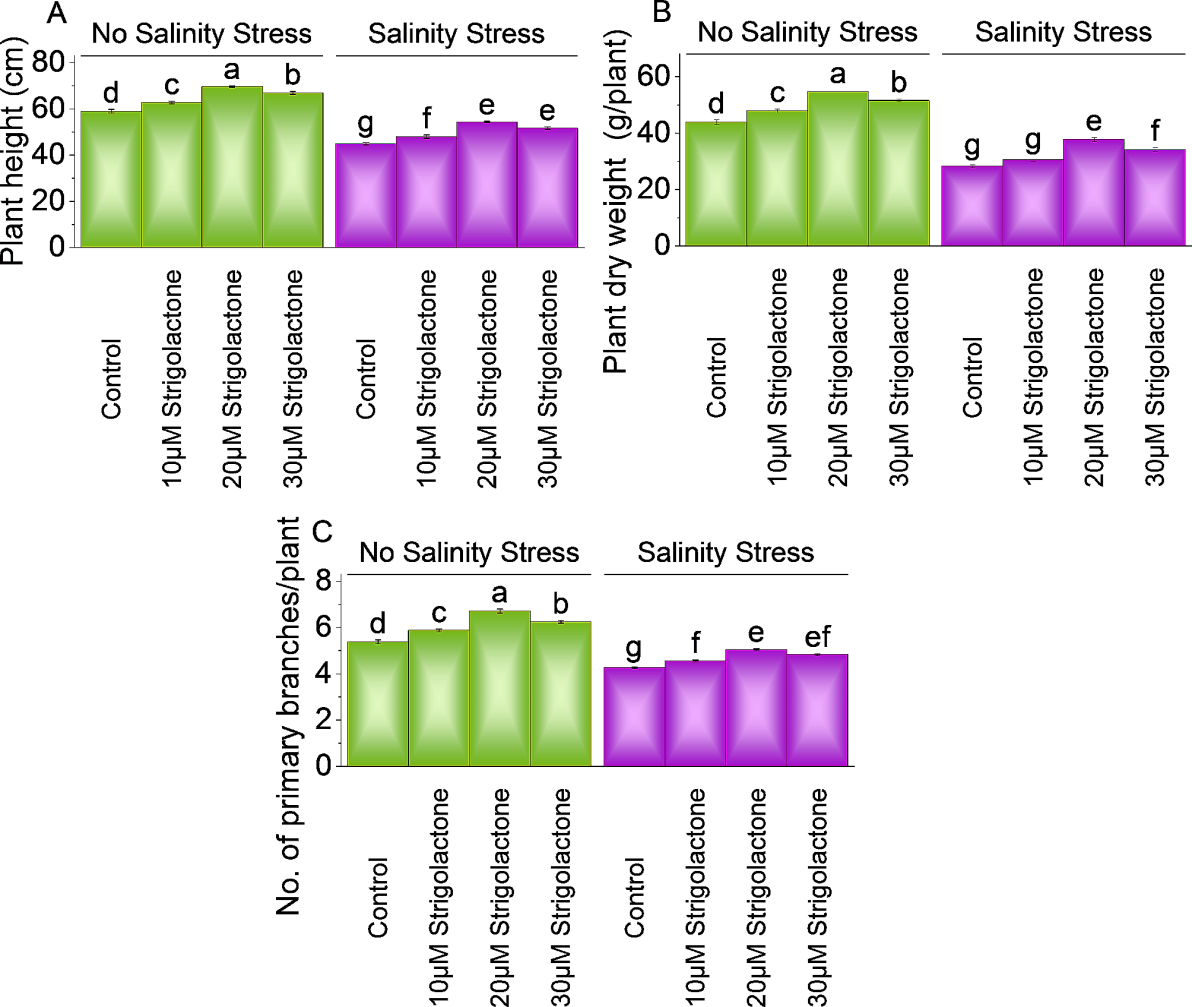
Effect of treatments on plant height **(A)**, plant dry weight **(B)** and number of primary branches **(C)** of chili under no salinity and salinity stress. The bars represent the means of four replicates with standard error. The Tukey test revealed significant changes at *p* < 0.05, shown by the different letters on the bars

Treatment 10µM strigolactone, caused elevation in plant height (6.38%) compared to the control under non-saline conditions. Applying 20µM strigolactone resulted in 18.11%, whereas 30µM strigolactone showed 13.40% increase in plant height than control under non-saline conditions. In salinity stress, 10µM strigolactone resulted in 6.82% increase in plant height compared to control. However, 20µM and 30 µM strigolactone caused 21.07 and 15.05% improvement in plant height than control under salinity stress (Fig. 1A).

In case of no salinity stress, 10µM strigolactone led to 9.03%, 20µM strigolactone exhibited 24.72% and 30µM strigolactone resulted in 17.54% improvement from control in plant dry weight. At salinity stress, 10µM strigolactone caused 8.09% increase in dry weight compared to control. In contrast, treatment 20µM strigolactone showed 33.60% while 30µM strigolactone caused 20.74% enhancement in dry weight under salinity stress (Fig. 1B).

In no-salinity stress, applying 10µM, 20µM and 30µM strigolactone treatment increase 9.19%, 24.65, and 16.06% in primary branches, over the control. Under salinity stress, 10µM strigolactone treatment increased 6.86%, 20µM strigolactone enhanced 18.17%, and 30µM strigolactone improved 13.25% in primary branches compared to control (Fig. 1C).

### Fruit length, girth, and yield

**Fig. 2:**
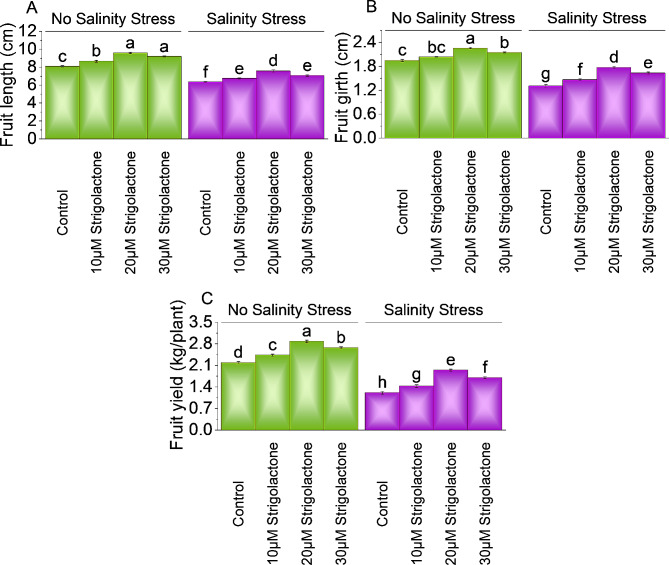
Effect of treatments on fruit length **(A)**, fruit girth **(B)**, and fruit yield **(C)** of chili cultivated under no salinity stress and salinity stress. The bars represent the means of four replicates with standard error. The Tukey test revealed significant changes at *p* < 0.05, shown by the different letters on the bars

Result showed that 10µM, 20µM and 30µM strigolactone treatment increased by ∼ 7%, ∼ 18%, and ∼ 14% in fruit length over control respectively under no salinity stress. In salinity stress, a significant increase in fruit length (∼ 6%, ∼ 19%, and ∼ 11%) was applied 10µM, 20µM and 30µM strigolactone treatment than control respectively (Fig. 2A).

Under no salinity stress applying 10µM, 20µM, and 30µM strigolactone treatment exhibited ∼ 5%, ∼ 16%, and ∼ 10% increases in fruit girth, respectively compared to control. Under salinity stress treatments with 10µM, 20µM, and 30µM strigolactone showed increases ∼ 12%, ∼ 35%, and ∼ 25% in fruit girth over control, respectively (Fig. 2B).

Under no salinity stress, a significant improvement in fruit yield by 11.20%, 31.43%, and 22.29% was applied 10µM, 20µM and 30µM strigolactone, over control respectively. At salinity stress, applying 10µM, 20µM and 30µM strigolactone increased the yield by 17.98%, 60.74% and 40.50% compared to the control (Fig. 2C).

### Chlorophyll contents

**Fig. 3:**
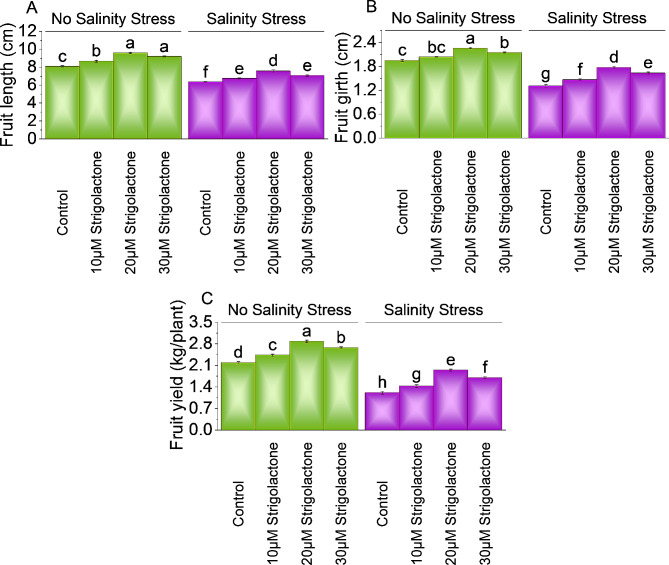
Effect of treatments on chlorophyll a **(A)**, chlorophyll b **(B)**, and total chlorophyll **(C)** of chili cultivated under no salinity stress and salinity stress. The bars represent the means of four replicates with standard error. The Tukey test revealed significant changes at *p* < 0.05, shown by the different letters on the bars

Chlorophyll-a content improved ∼ 6%, ∼ 15% and ∼ 11% were applied 10µM, 20µM, and 30µM strigolactone treatment, respectively compared to control under no salinity stress. In salinity stress, chlorophyll-a content significantly increased by 5.53%, 18.65% and 12.09%, were applied 10µM, 20µM and 30µM strigolactone treatments respectively over control (Fig. 3A).

In no salinity stress, treated with 10µM, 20µM, and 30µM strigolactone, chlorophyll-b content showed a ∼ 11%, ∼ 26%, and ∼ 17% increase above the control respectively. In case of salinity stress, applying 10µM strigolactone improved by ∼ 11%, 20µM strigolactone increased ∼ 44% and 30µM strigolactone enhanced ∼ 22% respectively than control (Fig. 3B).

The total chlorophyll content exhibited significantly increase ∼ 7%, ∼ 19%, and ∼ 13% with 10, 20, and 30µM strigolactone treatment over control under no salinity stress. Applying 10µM, 20µM, and 30µM strigolactone treatment increased by ∼ 7%, ∼ 25% and ∼ 15% in total chlorophyll content than control, respectively under salinity-stress (Fig. 3C).

### Electrolyte leakage (EL), Proline, and H_2_O_2_ content

**Fig. 4:**
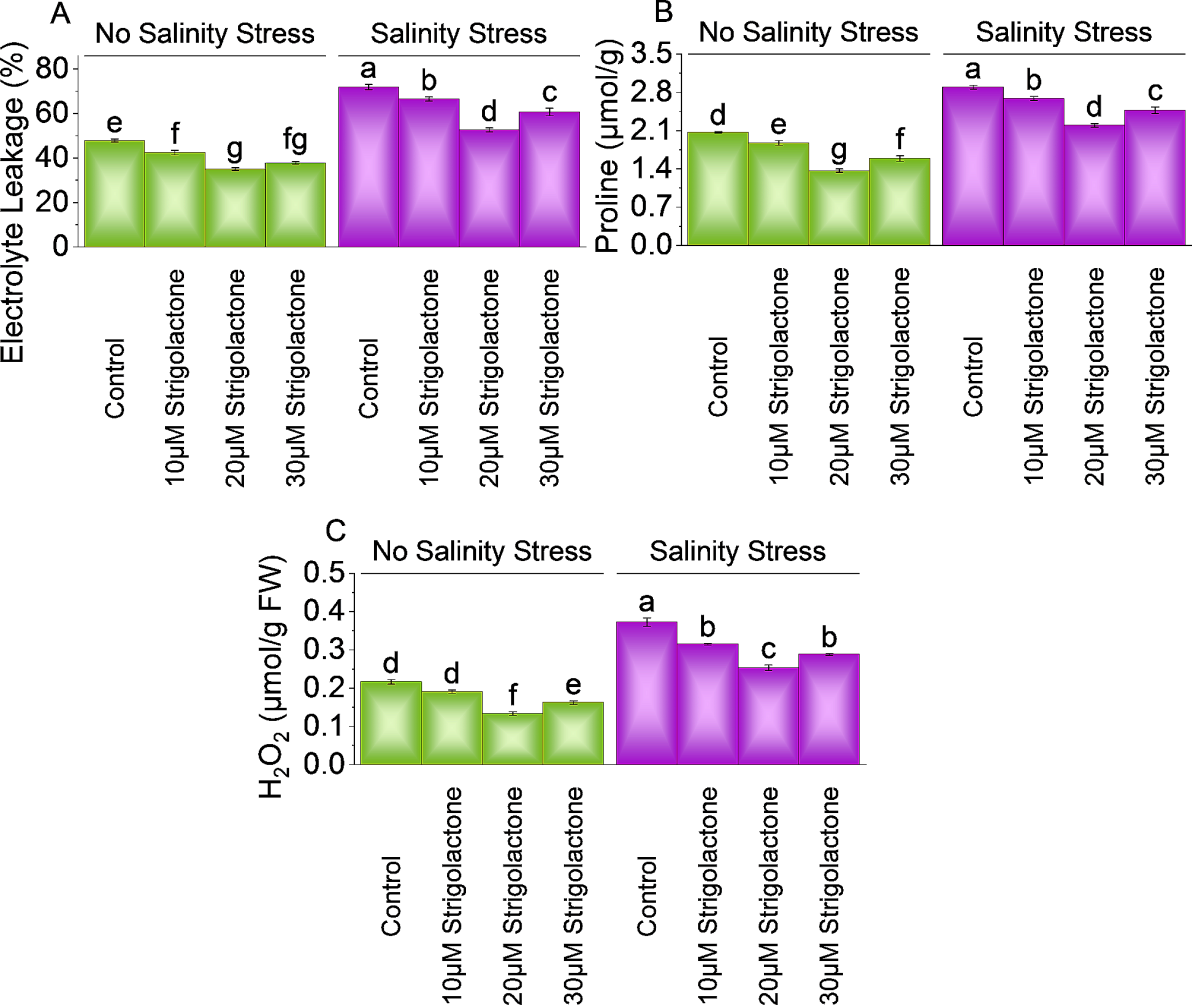
Effect of treatments on electrolyte leakage **(A)**, proline **(B)**, and H_2_O_2_**(C)** of chili cultivated under no salinity stress and salinity stress. The bars represent the means of four replicates with standard error. The Tukey test revealed significant changes at *p* < 0.05, shown by the different letters on the bars

The results showed that under no salinity stress ,10, 20 and 30µM strigolactones treatment decreased ∼ 13%, ∼ 37% and ∼ 27% by EL than control. In salinity stress, a significantly decreased EL by ∼ 8%, ∼ 37%, and ∼ 18%, were applied 10, 20 and 30µM strigolactones treatment, respectively over control (Fig. 4A).

Applying 10, 20 and 30µM strigolactone resulted ∼ 11%, ∼ 51% and ∼ 30% decrease in proline content over than control respectively under no salinity stress. Under salinity stress, the proline content significantly decreased by ∼ 8%, ∼ 32% and ∼ 17% with applying 10, 20 and 30µM strigolactones, above control respectively (Fig. 4B).

Under no salinity stress, treatment with 10, 20 and 30µM strigolactone resulted ∼ 14%, ∼ 62%, and ∼ 33% decrease in H_2_O_2_ levels over control respectively. In case of salinity stress, treatment with 10µM strigolactone exhibited decreased in ∼ 18%, 20µM strigolactone showed a ∼ 47%, and 30µM strigolactone resulted in ∼ 29% decrease respectively than control (Fig. 4C).

### MDA, SOD, and APX activity

**Fig. 5:**
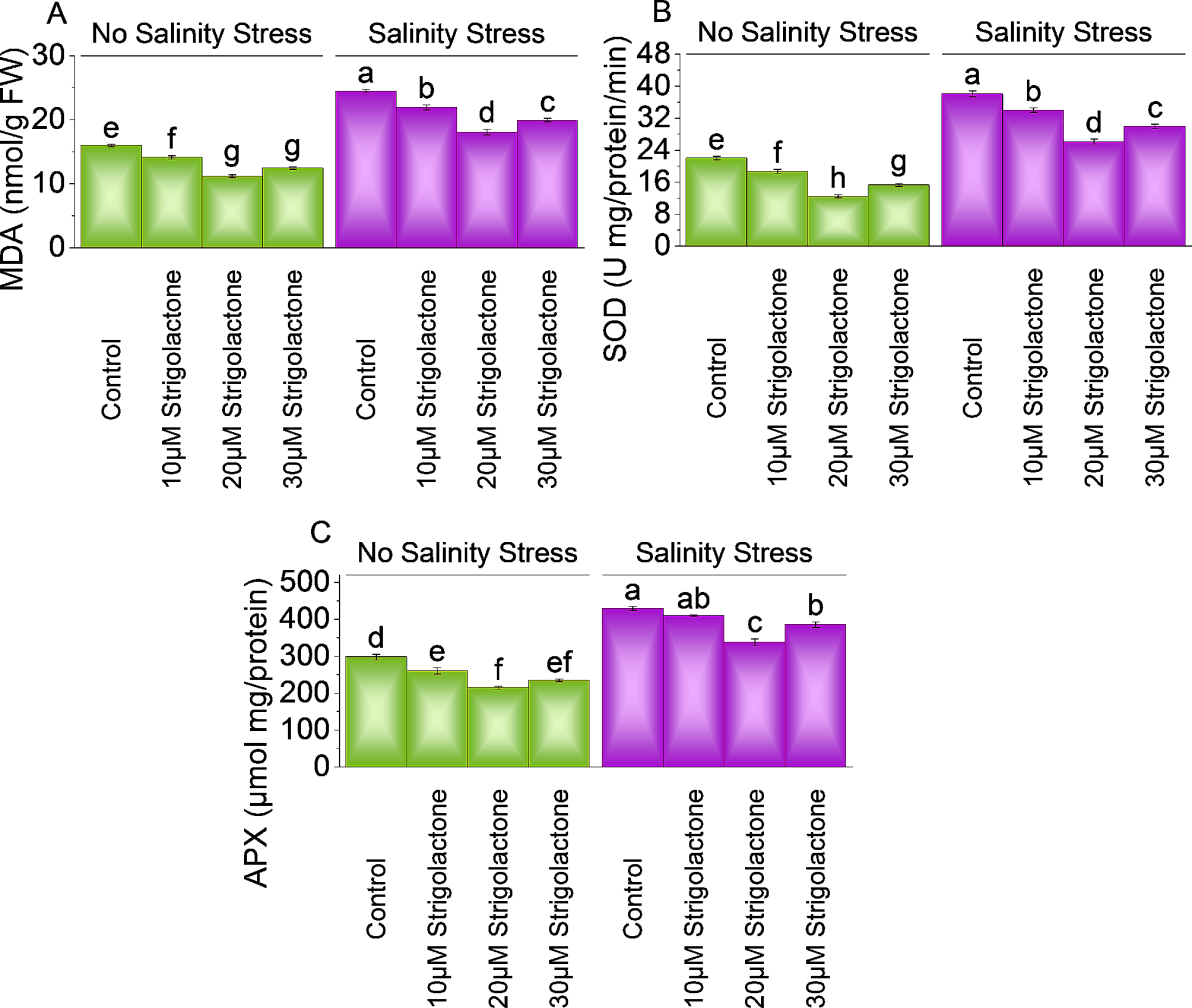
Effect of treatments on malondialdehyde (MDA) **(A)**, superoxide dismutase (SOD) **(B)**, and ascorbate peroxidase (APX) **(C)** of chili cultivated under no salinity stress and salinity stress. The bars represent the means of four replicates with standard error. The Tukey test revealed significant changes at *p* < 0.05, shown by the different letters on the bars

In no salinity stress, applying 10, 20 and 30µM strigolactone, MDA levels decreased by ∼ 13%, ∼ 43%, and ∼ 28% above control respectively. Under salinity stress, a significant decrease of MDA levels (∼ 12%, ∼ 35%, and ∼ 23%) were applied 10, 20 and 30µM strigolactone respectively than control (Fig. 5A).

Without salinity stress, applying 10, 20 and 30µM strigolactone significantly decreased SOD activity by ∼ 18%, ∼ 77%, and ∼ 44% over the control respectively. In salinity stress, SOD activity decline ∼ 12% at 10µM strigolactone, ∼ 46% at 20µM strigolactone and ∼ 27% at 30µM strigolactone respectively than control (Fig. 5B).

A significant decrease of APX level (∼ 15%, ∼ 39%, and ∼ 27%) was applied 10, 20 and 30µM strigolactone respectively than control under no salinity stress. In case of salinity stress, treatment with 10, 20 and 30µM strigolactone resulted ∼ 5%, ∼ 27% and ∼ 11% decrease in APX level compared to control (Fig. 5C).

### Leaves N, P, K, and na

**Fig. 6:**
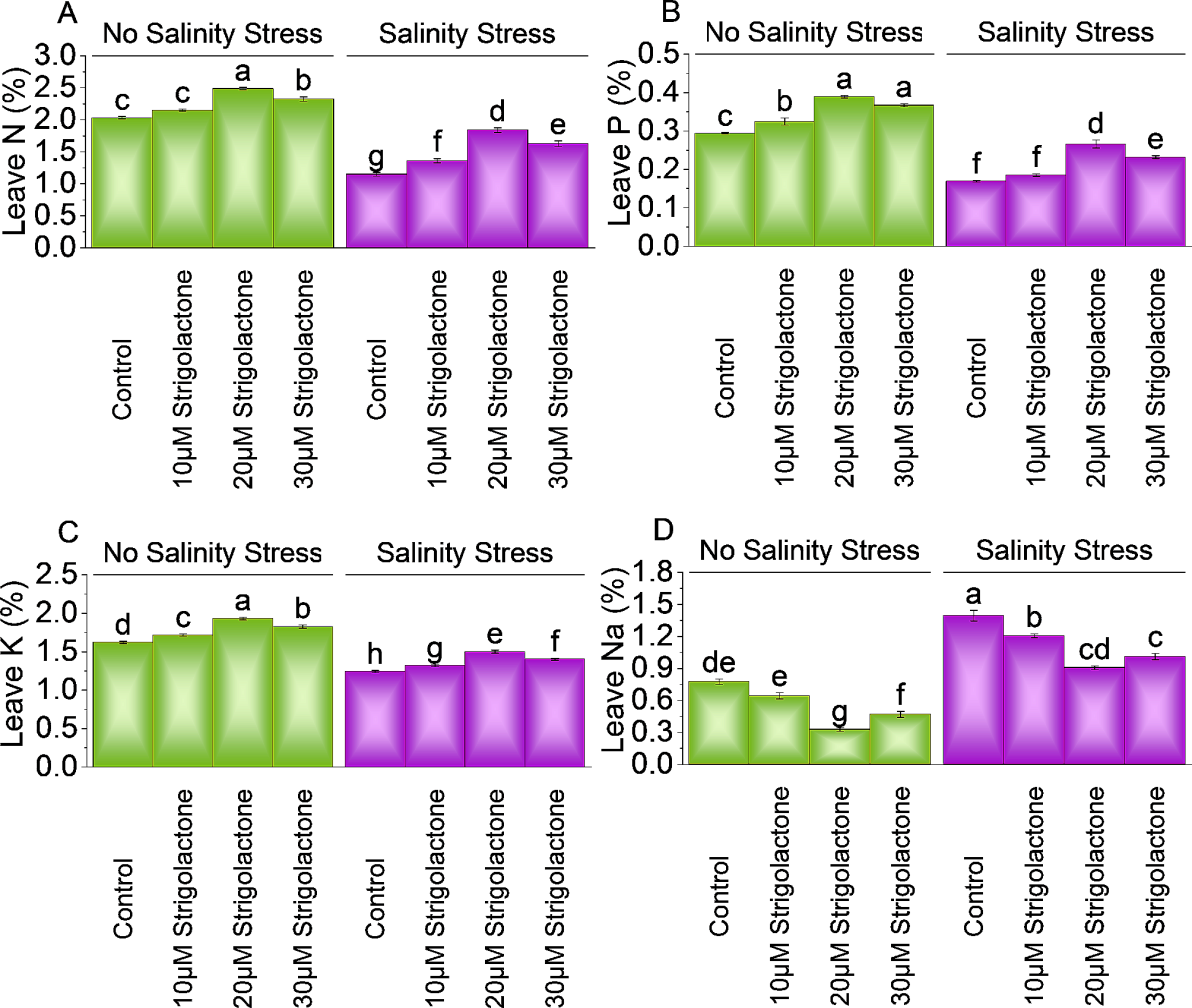
Effect of treatments on leaves N **(A)**, leaves P **(B)**, leaves K **(C)**, and leaves Na of chili cultivated under no salinity stress and salinity stress. The bars represent the means of four replicates with standard error. The Tukey test revealed significant changes at *p* < 0.05, shown by the different letters on the bars

At no salinity stress, applying 10, 20 and 30µM strigolactone resulted increases in leaves N content (∼ 6%, ∼ 22%, and ∼ 14%) compared to control respectively. Under salinity stress, a significantly increase leaves N content ∼ 18%, ∼ 60%, and ∼ 42% were applied 10, 20 and 30µM strigolactone treatment than control respectively (Fig. 6A).

In the absence of salinity stress, a significant enhancement of leaves *P* ∼ 10%, ∼ 32% and ∼ 25% with 10, 20 and 30µM strigolactone than control respectively. In salinity stress, treatment with 10µM strigolactone resulted ∼ 10%, 20µM strigolactone showed ∼ 57%, while 30µM strigolactone exhibited a ∼ 37% increase leaves P respectively over control (Fig. 6B).

Under no salinity stress, 10µM strigolactones increase K levels ∼ 6%, 20µM strigolactone caused ∼ 19%, and 30µM strigolactone enhanced ∼ 13% than control. In case of salinity stress, 10µM strigolactone exhibited ∼ 6% increase, in 20µM strigolactone improved ∼ 21%, while 30µM strigolactone enhanced ∼ 13% in leaf K levels compared to control respectively (Fig. 6C).

Applying 10, 20 and 30µM strigolactone resulted ∼ 21%, ∼ 137%, and ∼ 65% decrease in leaves Na, above control respectively no salinity stress. At salinity stress, a significantly decrease in leaves Na ∼ 16%, ∼ 54%, and ∼ 38% treatment with 10µM, 20µM and 30µM strigolactone than control respectively (Fig. 6D).

### Pearson correlation analysis

**Fig. 7:**
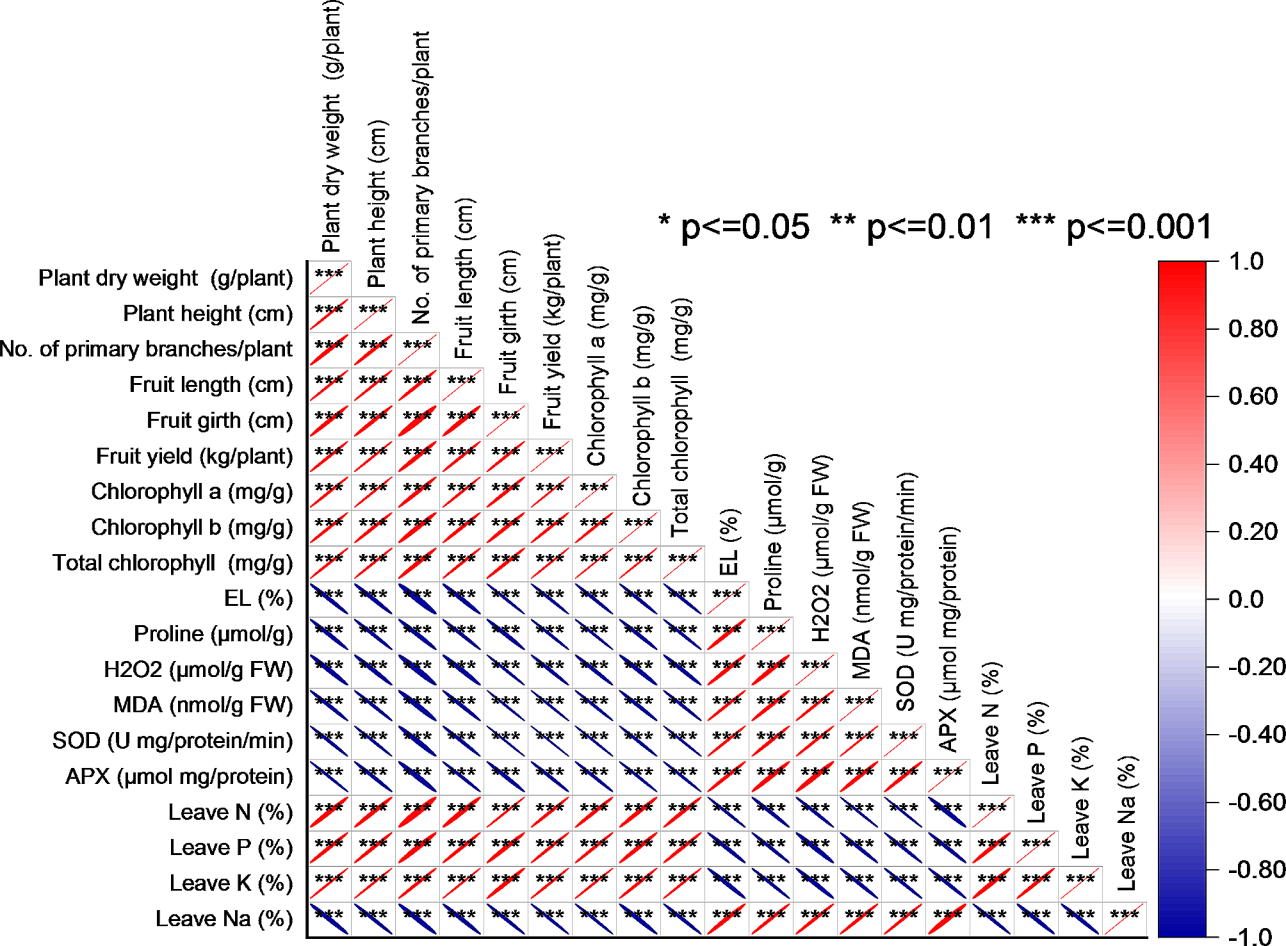
Pearson correlation for studied attributes

The pearson correlation analysis revealed strong relationships between various variables in the dataset. Plant dry weight exhibited a very high positive correlation of 1 with plant height, indicating that as plant height increased, the plant dry weight also increased significantly. Similarly, No. of primary branches/plant, Fruit length, fruit girth, fruit yield, chlorophyll a, and chlorophyll b displayed positive correlations with values close to 1, indicating a strong positive relationship. On the other hand, variables like EL, proline, H_2_O_2_, MDA, SOD, and APX exhibited strong negative correlations, suggesting an inverse relationship with the other variables. Interestingly, leaves N, leaves P, leaves K, and leaves Na also displayed correlations. Leaves N and leaves P had a positive correlation of approximately 0.985, while leaves K and leaves Na exhibited positive and negative correlations with values around 0.997 and − 0.986, respectively, suggesting interdependencies between these variables (Fig. 7).

## Discussion

### Morphological attributes

Strigolactone exhibit a range of morphological effects. It can mitigate salinity stress by helping maintain ion homeostasis in plant roots and improving water and nutrient acquisition, making them valuable in challenging growing conditions [37]. Strigolactone can also stimulate shoot elongation by modulating auxin transport, leading to increased plant height with higher concentrations of strigolactone causing more significant height increments [38]. Moreover, they enhance dry weight and root development via better nutrient uptake and increased biomass production [39]. Furthermore, strigolactone promotes branching by inhibiting the outgrowth of axillary buds, increasing the number of primary branches, a desirable trait in agriculture for potential fruit production [40].

### Chlorophyll contents

The application of strigolactone results in augmented chlorophyll content, particularly chlorophyll a and b. This elevation is linked to their role in positively regulating genes associated with photosystem II (PSII) and light-harvesting complex (LHC) proteins [41, 42]. Furthermore, strigolactone help mitigate stress-induced chlorophyll degradation and regulate stomatal conductance, thereby improving water use efficiency and maintaining higher chlorophyll levels under stress conditions [43].

### Electrolyte leakage

In their role of mitigating electrolyte leakage under salinity stress, Strigolactone likely exert their influence through intricate gene regulatory networks and pathways. Studies suggest their potential modulation of genes involved in membrane stability, such as lipid desaturases or proteins linked to membrane repair mechanisms [44, 45]. Additionally, strigolactone might impact osmotic adjustment pathways, potentially regulating genes related to proline biosynthesis (e.g., P5CS) or sugar synthesis to aid in cellular osmotic regulation and prevent water loss-induced membrane damage [9, 46].

### Nutrient - potassium

Potassium plays a pivotal role in plant physiology, aiding in osmotic regulation by maintaining turgor pressure to prevent wilting, activating enzymes for crucial metabolic processes, facilitating nutrient uptake, regulating stomatal function for improved photosynthesis and reduced transpiration, and mitigating the effects of abiotic stress like salinity, promoting healthier growth, as seen in chili plants treated with strigolactone [47, 48].

### Antioxidants

Strigolactone plays a crucial role in enhancing a plant’s antioxidant defense system by inducing the expression of stress-responsive genes like superoxide dismutase (SOD) and ascorbate peroxidase (APX) [49]. This augmentation in antioxidant activity helps in reducing oxidative stress, particularly under unfavorable conditions. Additionally, by modulating hormonal balance, specifically abscisic acid (ABA) and jasmonic acid (JA) levels, strigolactone contributes to the plant’s stress response mechanisms [46, 50]. Studies suggest their influence on genes associated with proline biosynthesis, notably those encoding enzymes like pyrroline-5-carboxylate synthetase (P5CS), a key enzyme in proline biosynthesis. Strigolactone might modulate the expression or activity of P5CS and other genes involved in proline metabolism, leading to increased proline accumulation [17]. Proline acts as a vital osmoprotectant, aiding cellular osmotic adjustment and maintaining cell turgor under saline conditions [51].

## Conclusion

In this study, it is evident that the 20µM strigolactone holds the potential for enhancing chili growth. This treatment exhibits the capacity to improve chlorophyll content and nutrient uptake in leaves, particularly in essential elements like phosphorus (P), nitrogen (N), and potassium (K), which are essential for chili growth. Additionally, the 20µM strigolactone treatment demonstrates the potential to effectively regulate antioxidant mechanisms. Further research at the field level is recommended to validate the efficacy of this treatment in promoting chili growth under salinity stress.

## Data Availability

All data generated or analysed during this study are included in this published article.

## Declarations

### Experimental research and field studies on plants (either cultivated or wild), including the collection of plant material, must comply with relevant institutional, national, and international guidelines and legislation

We confirmed that all methods were performed in accordance with the relevant guidelines / regulations / legislation.
